# Influence of the Addition of Dispersible Color Powder and Polyacrylic Emulsion on the Durability of Cement Mortar

**DOI:** 10.3390/ma15155305

**Published:** 2022-08-02

**Authors:** Chih-Ming Huang, Her-Yung Wang, Wein-Duo Yang, Tien-Chun Kao, Sing-Yuan Fang

**Affiliations:** 1 Department of Chemical and Materials Engineering, National Kaohsiung University of Science and Technology, Kaohsiung City 807, Taiwan; color.jackhuang@gmail.com (C.-M.H.); v27946@gmail.com (S.-Y.F.); 2 Department of Civil Engineering, National Kaohsiung University of Science and Technology, Kaohsiung City 807, Taiwan; wangho@nkust.edu.tw (H.-Y.W.); asd75986417@gmail.com (T.-C.K.)

**Keywords:** inorganic color powder, polyacrylic emulsion, color mortar, durability, surface electrical resistivity, thermal conductivity

## Abstract

Cement mortar can be colored using color additive technology to give colorful facades to the surfaces of buildings, and to beautify the environment. In this study, weight ratios of color powder/cement at 1:80, 1:40, and 1:27, and polyacrylic emulsion/cement at a ratio of 1:5 were added as pigments to cement mortar; the fresh properties, slump, slump flow, hardened properties, compressive strength, flexural strength, ultrasonic pulse velocity, durability, surface electrical resistivity and thermal conductivity of the colored cement mortar were then examined. The results showed that adding color powder/cement at 1:80 and polyacrylic emulsion/cement at 1:5 gives the best water/cement (W/C) ratio, which equals 0.5; this can effectively improve the hardness and durability of colored cement mortar. At 28 days of aging, the strength of the various colored cement mortars was maintained at 33.1–36.8 MPa. The acrylic-based emulsion significantly improved the flexural strength of the specimen. At 91 days of aging, all of the cement mortars exceeded the control group, with an anti-bay strength of 19.9–21.7 MPa, and the strength increased with aging. Adding appropriate amounts of inorganic color powder and mixing water can effectively enhance the fresh and hardened properties and durability of the colored cement mortar, while polyacrylic emulsion may significantly improve the test pieces and flexural strength, which increases with age. Moreover, natural α-Fe_2_O_3_ (rust layer) is formed on the surface of the colored cement mortar samples through the addition of inorganic color powder that contains Fe(III) ion; this prevents the intrusion of noxious ions and thus increases the durability. All of the test pieces of colored cement mortar in this study had a surface resistance of over 20 kΩ-cm on the seventh day of the test period, meaning good surface compactness. In addition, because the thermal conductivity of the added inorganic color powder was higher than that of cement, the thermal conductivity was significantly improved.

## 1. Introduction

The construction industry has experienced a year-on-year escalating demand for building materials, which puts a burden on the environment. About 10% of CO_2_ emissions worldwide come from building materials, and cement accounts for about 85% [1] of it. As time passes, many timeworn residences and buildings in Taiwan fail to live up to the current regulations and safety standards. Antiquated residences create safety concerns, and so must be overhauled or renovated [2]. In recent years, Taiwan has been troubled by the problems of a persistent low birth rate and labor shortages without seeing improvement or mitigation, resulting in difficulty in the development and transformation of the construction industry [3]. In order to solve the problem concerning labor demand and protect the environment by reducing CO_2_ emissions, the industry has to address the time and labor-consuming work procedures of traditional exterior wall processing. Colored cement is an effective solution. Furthermore, the sustainability requirements for cement-based materials, such as the reduction of CO_2_ emissions and concerns about other environmental impacts, make the large-scale use of solid wastes and the development of highly durable concrete necessary. In the construction industry, cement is an important material in reinforced concrete (RC) structures, and is widely used. However, the appearance of buildings is mostly dark gray after casting, and paint or other coatings must be used to improve this appearance. In order to solve this problem, through the colorization of cement mortar, the effect of beautification and environmental protection is achieved through technologies such as color additives; thus, the surface of the building structures can directly show the appearance of color.

Colored cement mortar is a special concrete material to which color additives can be applied. Concrete buildings’ appearance or structural surfaces are directly exposed by the use of the techniques of formwork, pouring and curing in order to give special colors to building structures for beautification [4]. Different pigments [5] are used to provide different colors of cement mortar. Either inorganic mineral pigments or organic polymer can be used as pigments for dyeing. Commonly used inorganic mineral pigments include iron oxide, titanium oxide and chromium oxide [6]. Compared to organic polymer pigments, inorganic mineral pigments are more durable in the general environment, and thus are more widely used [7]. In 2017, Kappel et al. replaced some of the cement mortar with sewage sludge ash (SSA) and dyed it; they then examined the color, compressive strength and workability [8]. In 2018, Zhang et al. tried to reuse harmful fly ash (FA) by adding it to the cement mortar; they found that the color variations after thermal processing could serve as an indicator of changes in moisture content [9]. In 2019, Aalil et al. used locally recycled brick waste to make pozzolanic mortar. They found that the lightness (L*) of colors was reduced when brick waste was added, and the red–green a* and yellow–blue b* parameters increased, affecting the color presentation of the mortar [10]. In 2020, López et al. dyed portland cement with the following pigments: ferric oxide yellow, red, and black, and copper phthalocyanine blue and green. They then defined the color variations using CIELAB color space and the color-difference formula [11]. In 2021, Zheng et al. used copper slag, an industrial waste product, to modify the color of cement mortar. A total of 40 wt.% content of the copper slag used was ferric oxide, the same as that used in the inorganic mineral pigment for making colored cement mortar, while the remaining 60 wt.% was gel-active silica and alumina minerals. The research results showed that the best compressive strength (7.33 MPa) and flexural strength (3.01 MPa) can be achieved by adding 10 wt.% copper slag [12]. Using recycled wastes to make colored cement mortar is an excellent method of ensuring environmental friendliness, but quality control can be a challenge. Few studies have been conducted on the use of pigments for colored cement mortar, and further discussion on the nature of the construction works is needed.

However, in traditional construction projects, the appearance of most completed buildings is gray or dark, and paint, tiles and other decorative materials are often required to improve the appearance. There is no substantial solution in terms of material cost and environmental protection issues. Since the end of the 19th Century, metal oxides have been added to cement mortar as a method of wall modification [13]. The facade of the City of Justice in Barcelona, Spain, in 2002 was completed with colored cement mortar [14]. In addition to direct dyeing in the cement mortar to improve appearance conveniently, the addition of colorants also affects the fluidity and engineering properties, and may even change the process of the cement hydration reaction. Previous research has determined that metal oxide inorganic pigments have excellent performance in heat and light, alkali resistance, and durability, and have been widely used in cement products [15].

In the past, inorganic pigments were mostly obtained from nature; for example, hematite and ochre ores of iron oxide were often used as inorganic pigments for red and yellow [16,17]. Most modern pigment production is carried out using synthetic methods, such as the use of chromium oxide [18]. However, there are few reports on the influence of the color stability, particle size and amount of the pigment added to the cement mortar.

Accordingly, the aim of this study was to add colors to ordinary cement mortar by precisely using inorganic color powder pigments (red, yellow, green, blue, white and black), while polyacrylic emulsion was also added to examine its influence on colored cement mortar. Furthermore, the composition of the pigments for colored cement mortar was examined; along with the changes with different quantities of added pigments, different water/cement ratios and different ages of growth were also analysed. Microanalysis was performed with SEM imaging to further study the fresh properties, hardened properties and durability of colored cement mortar.

## 2. Research Materials and Methods

### 2.1. Research Materials

**Figure 1:**
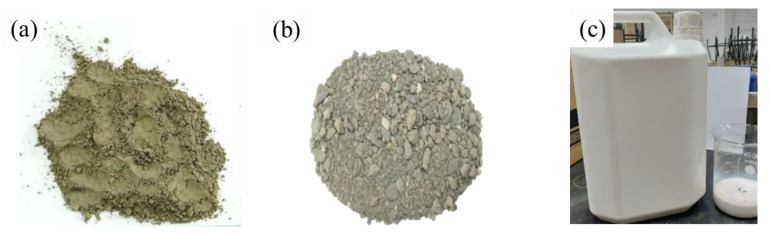
Materials: (**a**) cement, (**b**) fine aggregate, and (**c**) polyacrylic emulsion.

**Figure 2:**
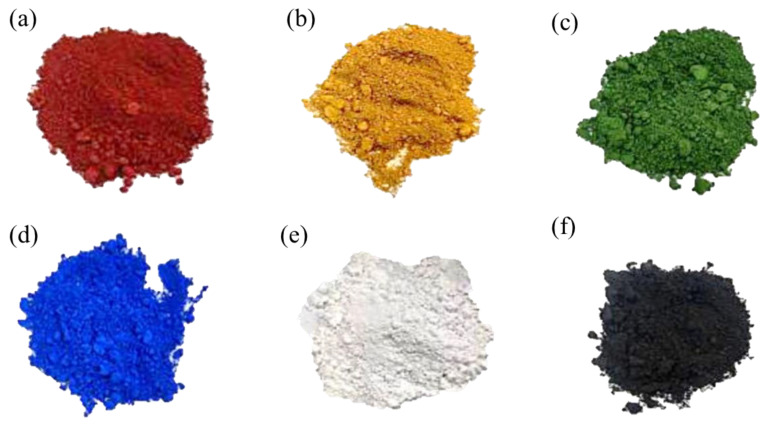
Inorganic color powders: (**a**) red, (**b**) yellow, (**c**) green, (**d**) blue, (**e**) white, and (**f**) black.

The materials used in this study include cement, nano-inorganic color powder, polyacrylic emulsion and fine aggregate. The details of the research materials are listed below:

(1) Cement: Portland cement type I produced by Taiwan Cement Corporation (Taipei, Taiwan) was used in this study, which conforms to the ASTM C150 standard [19], with a specific gravity of about 3.15, as shown in Figure 1a.
(2) Fine aggregate: Natural river sand from the Ligang River was used, conforming to the ASTM C33 standard [20], with a specific gravity at 2.65 and water absorption of 2.0%, as shown in Figure 1b.
(3) Mixing water conforming to the ASTM C1602 standard [21] was used.
(4) The polyacrylic emulsion was provided by Tai Chang Resin Industrial Co., Ltd. (Tainan, Taiwan), as shown in Figure 1c, with the basic properties shown in Table 1.
(5) The inorganic color powder was provided by LANXESS Chemical Co., Ltd. (Beijing, China), as shown in Figure 2; its basic properties are shown in Table 2.

### 2.2. Test Variables and Mix Proportions

The proportions of mixing water used in this study for mixing with the inorganic color powder and polyacrylic emulsion at different water/cement ratios (0.4, 0.5 and 0.6) are shown in Table 3, below.

### 2.3. Test Items and Methods

Slump was measured according to the ASTM C143 standard [22]. Slump flow was measured according to the ASTM C230 standard [23]. Compressive strength was measured according to the ASTM C109 standard [24]. Flexural strength was measured according to the ASTM C348 standard [25]. Ultrasonic pulse velocity was measured according to the ASTM C597 standard [26]. Surface resistivity was measured according to the ASTM C876 standard [27]. Thermal conductivity was measured according to the ASTM E1225 standard [28]. Regarding the FE-SEM analysis, the test samples’ surfaces were observed using FE-scanning electronic microscopy (FE-SEM, AURIGA, JEOL6330) for morphology analysis. The test samples were meticulously cut into cubes of 8 × 8 × 8 mm for the FE-SEM observation. Because the test samples are non-conductive, they were metal-plated before analysis.

## 3. Results and Discussion

### 3.1. Fresh Properties

**Figure 3:**
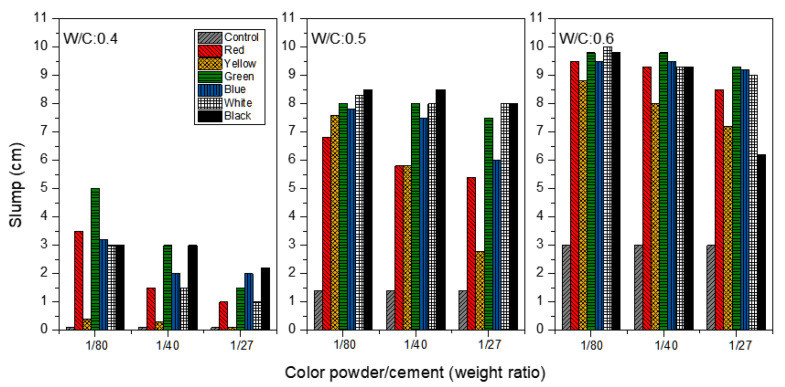
Slump of colored mortar at W/C = 0.4, 0.5 and 0.6, with weight ratios of color powder/cement at 1:80, 1:40, 1:27 added.

As seen in Figure 3, colored cement mortar was observed for the slump analysis at the W/C ratios of 0.4, 0.5 and 0.6, with the addition of color powder/cement at 1:80, 1:40, 1:27. The slump values of the control group mortar, with no inorganic color powder added, at W/Cs of 0.4, 0.5 and 0.6 are 0.1, 1.4 and 3.0 cm, respectively. This means that the slump value of the mortar increases as the W/C rises. When the W/C equals 0.4, it is shown clearly that the slump decreases as inorganic color powder is increased from 1:80 to 1:27 because nano-inorganic color powder absorbs some of the mixing water due to its porosity; thus, the slump value generally declines as more color powder is added. In particular, the slump value of cement mortar with yellow color powder decreases from 0.4 to 0.1 cm, which is nearest to the control group’s slump (0.1), when the yellow powder is increased from 1:80 to 1:27. This result is probably attributable to the large surface area (63.4 m^2^/g) of the yellow powder; the internal friction of the particles tends to lower the slump value as the number of color powder particles increases [29]. Furthermore, it can be seen that the slump values of mortar generally increase when W/C equals 0.5 and 0.6, probably because a higher W/C ratio supplies the much-needed mixing water for the colored mortar. At the W/C ratios of 0.5 or 0.6, the impact of the mixing water on the slump is greater than that of the color powder additive.

**Figure 4:**
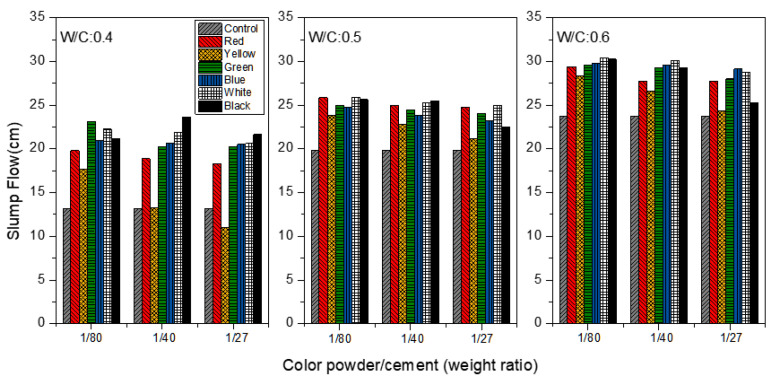
Slump flow of colored mortar at W/C = 0.4, 0.5 and 0.6 when weight ratios of color powder/cement at 1:80, 1:40, and 1:27 are added.

Figure 4 shows the slump flow analysis of the mortar at the W/C ratios of 0.4, 0.5 and 0.6, respectively, when color powder/cement ratios at 1:80, 1:40, 1:27 are added. In the control group to which no color powder was added, the slump flow values of mortar are 13.15, 19.83 and 23.70 cm when W/C = 0.4, 0.5 and 0.6, respectively, meaning that the inorganic color powder has only a limited impact on the slump flow, and a greater influence on the workability of mortar is seen at higher W/C ratios. For example, at the W/C ratio of 0.4, it can be seen that the slump flow of yellow cement mortar is 11.00 cm when 1:27 yellow powder is added, which is smaller than the slump flow of the control group (13.15 cm). This means that when yellow inorganic color powder that has large surface area is added, the high content of powder particles tends to increase the internal friction of the particles, leading to a lower slump flow value.

### 3.2. Hardened Properties

**Figure 5:**
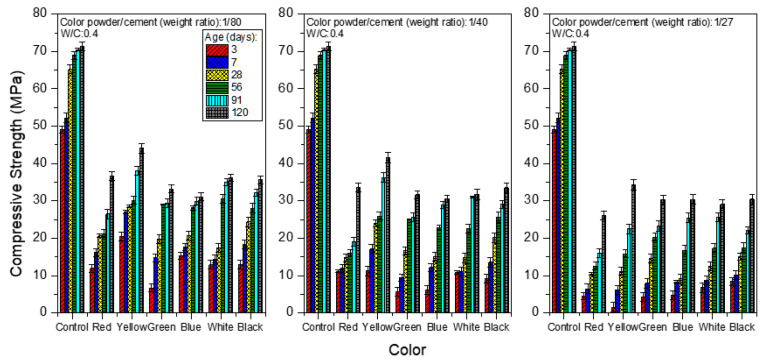
Compressive strength of colored mortar at W/C = 0.4 and under saturated limewater curing when weight ratios of color powder/cement at 1:80, 1:40, and 1:27 are added.

Figure 5 shows the compressive strength analysis of the mortar obtained from the weight ratios of color powder/cement at 1:80, 1:40 and 1:27, respectively, at the W/C ratio of 0.4, and under saturated limewater curing. The compressive strength of the mortar increases with age. The compressive strength values of the control group mortar are 49.07, 65.19 and 71.30 MPa at day 3, day 28 and day 120, respectively, representing about a 24.73% increase in compressive strength from days 3 to 28, and about a 9.37% increase in compressive strength from days 28 to 120. It is discernible that the compressive strength of colored mortar obtained from the weight ratio of polyacrylic emulsion/cement at 1:5 with color powder is consistently smaller than that in the control group at any age, and the greater the amount of color powder added, the lower the compressive strength, which may be due to the aggregation and uneven spread of color powder particles in the mortar when more powder is added, which leads to the formation of weak zones [30]. This may also cause the formation of cracks in the blocked air voids when the cement mortar ages [31].

**Figure 6:**
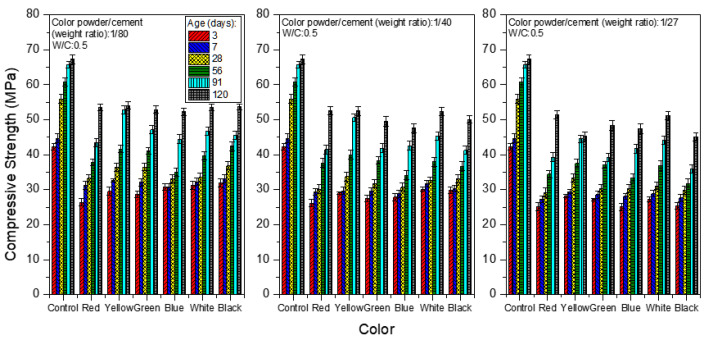
Compressive strength of colored mortar at W/C = 0.5 and under saturated limewater curing when weight ratios of color powder/cement at 1:80, 1:40, and 1:27 are added.

**Figure 7:**
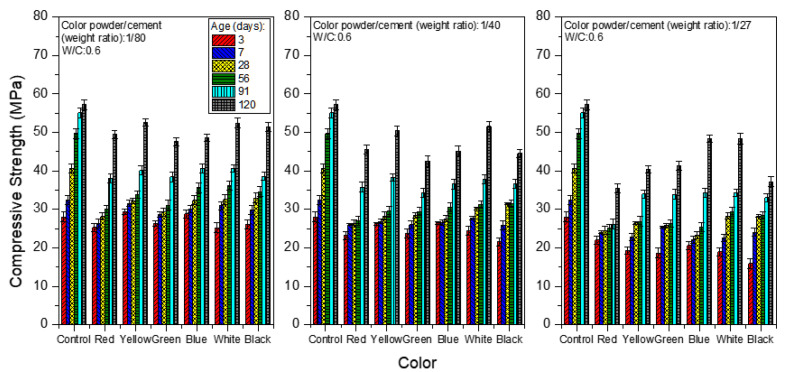
Compressive strength of colored mortar at W/C = 0.6 and under saturated limewater curing when weight ratios of color powder/cement at 1:80, 1:40, and 1:27 are added.

Figure 6 and Figure 7 show the compressive strength when the W/C equals 0.5 and 0.6, respectively. Among all of the test pieces of the colored mortar with added polyacrylic emulsion and inorganic color powder, those with W/C = 0.5 show a better compressive strength compared to mortar with other water/cement ratios. Taking the mortar obtained by aging for 28 days as an example, when the weight ratio of color powder/cement is 1:80, the compressive strength is 17.4–28.5 MPa; when the color powder/cement ratio is 1:40, the compressive strength is 14.7–23.9 MPa, and when the color powder/cement ratio is 1:27, the compressive strength is 9.12–15.1 MPa. A possible reason is the porous inorganic color powder absorbing some of the mixing water, such that less water is available for the hydration reaction in the process. This explains why the strength at W/C = 0.4 is lower than that at W/C = 0.5, whereas at W/C = 0.6, the porosity increases because there is more free water, leading to a lower compressive strength.

**Figure 8:**
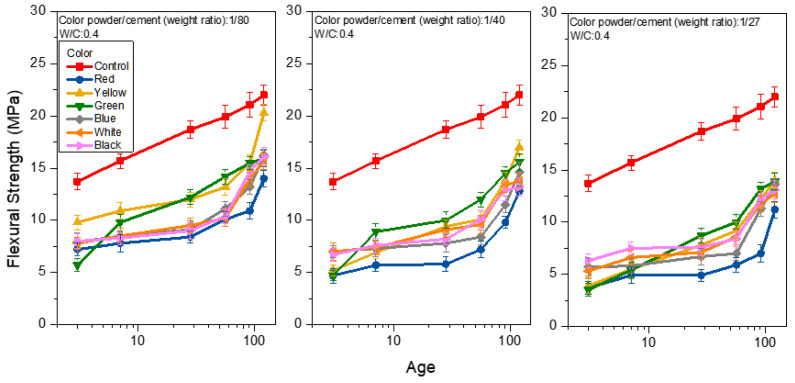
Flexural strength of colored mortar at W/C = 0.4 and under saturated limewater curing when weight ratios of color powder/cement at 1:80, 1:40, and 1:27 are added.

**Figure 9:**
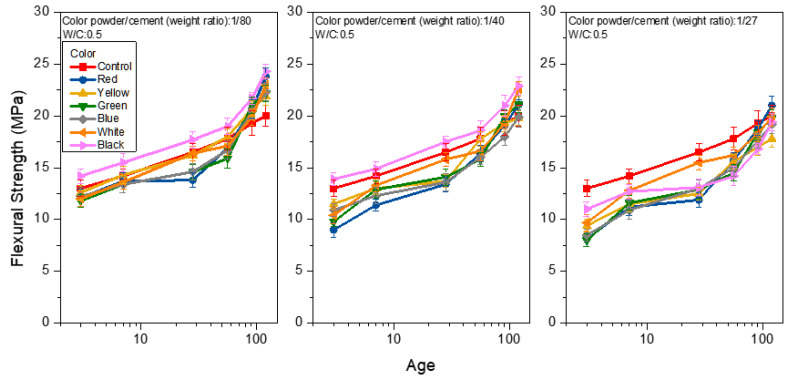
Flexural strength of colored mortar at W/C = 0.5 and under saturated limewater curing when weight ratios of color powder/cement at 1:80, 1:40, and 1:27 are added.

**Figure 10:**
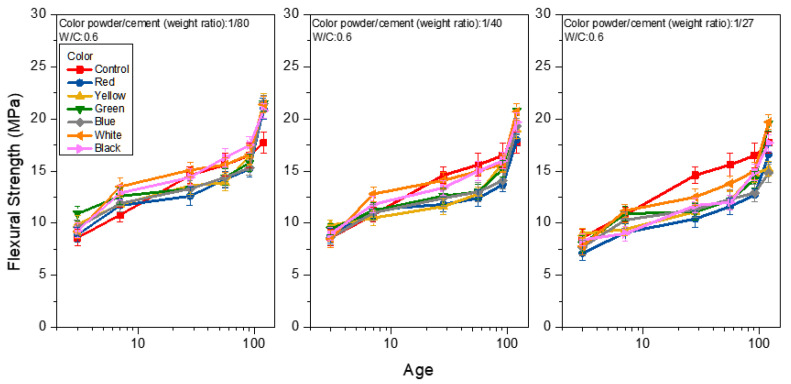
Flexural strength of mortar at W/C = 0.6 and under saturated limewater curing when weight ratios of color powder/cement at 1:80, 1:40, and 1:27 are added.

Figure 8, Figure 9 and Figure 10 show the flexural strength analysis of the mortar prepared from weight ratios of color powder/cement at 1:80, 1:40, and 1:27, respectively, at the W/C ratios of 0.4, 0.5 and 0.6, and under saturated limewater curing. The flexural strength of the control group was 22.0, 20.0 and 17.7 MPa at the 120-day age of each W/C ratio (0.4, 0.5, 0.6). With the increase in the W/C ratio, the flexural strength decreased. This can be seen from Figure 8 when W/C equals 0.4 and the weight ratios of color powder/cement are 1:80, 1:40, and 1:27, respectively. The flexural strengths of the mortar at 120 days are 14.3–20.3, 12.8–17.0 and 11.2–14.0 MPa, respectively; the flexural strength decreases with the increase in of the color powder’s addition. The same explanation for the compressive strength test results also applies here. Because the inorganic color powder absorbs some of the mixing water, the reduced amount of water in the hydration process of colored mortar weakens the strength. It is noteworthy that at the W/C ratio of 0.5 and the color powder/cement weight ratio = 1:80 (as shown in Figure 9), the flexural strength exceeds that of the control group at 91 days. At 120 days, and when the flexural strength reaches 21.9–24.3 MPa, the flexural strength of mortar obtained from color powder/cement weight ratios of 1:40 and 1:27 is about 19.8–22.9 MPa and between 17.8–21.0 MPa, respectively. In addition, when the W/C is 0.6 at 120 days (as shown in Figure 10), the flexural strength analysis results are similar to those of W/C at 0.5, and the flexural strength of some colored cement mortars is close to or greater than that of the control group. In addition, when the mortar is prepared with a W/C of 0.6 at 120 days (as shown in Figure 10), the flexural strength analysis results are similar to those of W/C at 0.5, and the flexural strength of some of the colored cement mortars is close to or greater than that of the control group. The hydration reaction reaches a higher level with age, and the solidified polyacrylic emulsion ensures considerable ductility that enhances flexural strength.

**Figure 11:**
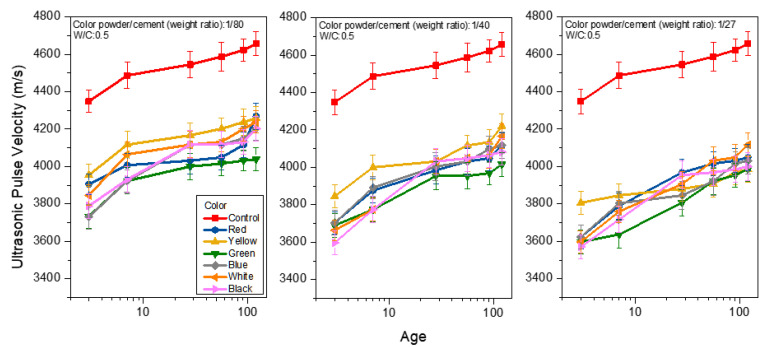
Ultrasonic pulse velocities of colored mortar with different proportions of inorganic color powder.

The air voids and cracks inside the colored mortar samples were reviewed using ultrasonic pulse velocity (UPV) analysis. Table 4 and Figure 11 show the UPV analysis of the mortar at the W/C ratio of 0.5 and under saturated limewater curing when weight ratios of color powder/cement at 1:80, 1:40, and 1:27 were added. The UPV values of the control group were 4348, 4545 and 4557 m/s at day 3, day 28 and day 120, respectively, meaning that the UPV increases with age. There was a 4.33% growth in UPV from days 3 to 28, and a 2.46% growth from days 28 to 120. It is noticeable that the UPV values of the colored cement mortar samples with added 1:80 inorganic color powder ranged between 3731 and 3954 m/s at day 3, between 4000 and 4167 m/s at day 28, and between 4202 and 4269 m/s at day 120. It was found that at any given age, all of the samples have smaller UPV values than the control group. The UPV is also lowered with more inorganic color powder added, which means that there are more air voids inside the samples; this is consistent with the compressive strength analysis results and the possible explanation mentioned above [30,31]. Moreover, all of the samples were added to a weight ratio of polyacrylic emulsion/cement at 1:5. Although solidified emulsion augments the samples’ flexural strength, polyacrylic emulsion is prone to the generation of air bubbles when mixed with cement mortar, and the air cannot be effectively expelled through compression [4]. Therefore, all of the colored mortar samples had smaller UPV values than the control group.

### 3.3. Durability

**Figure 12:**
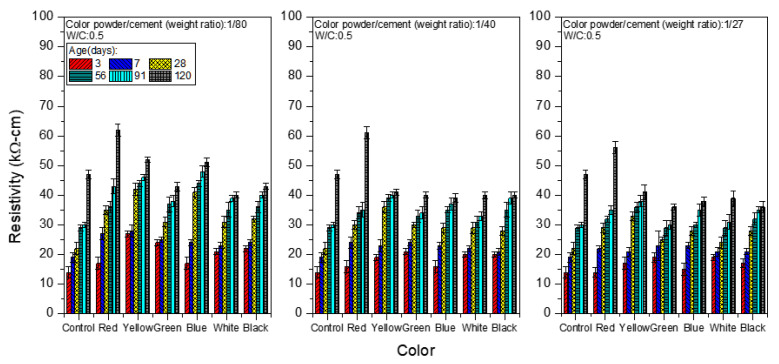
Surface electrical resistivity of colored mortar with different proportions of inorganic color powder.

Surface electrical resistivity analysis may serve as a non-destructive inspection method that indicates the durability of cement mortar [32]. Table 5 and Figure 12 show the surface electrical resistivity analysis of colored mortar at the W/C ratio of 0.5 when weight ratios of color powder/cement at 1:80, 1:40, and 1:27 were added. The surface electrical resistivity values of the control group at different ages (day 3, day 28 and day 120) are 14, 22 and 47 kΩ-cm, respectively. Adding polyacrylic emulsion and inorganic color powder increased the surface electrical resistivity of all of the samples, probably because the nanoparticles of inorganic color powder can effectively block the intrusion and corrosion of ions in the water. When the colored cement mortar samples age and the hydration process gradually completes, enhancing the mortar’s compactness, the surface electrical resistivity increases. For example, at day 28 with 1:80 inorganic color powder, the sample’s surface electrical resistivity is 31–42 kΩ-cm, while it is 28–36 kΩ-cm with 1:40 inorganic color powder and 24–33 kΩ-cm with 1:27 inorganic color powder. As more inorganic color powder is added, the surface electrical resistivity drops slightly, probably because excessive porous inorganic color powder creates more pores inside the samples, thus lowering the surface electrical resistivity, which corresponds to the results of the UPV analysis above.

In particular, red colored mortar samples have the highest surface electrical resistivity, followed by yellow colored mortar, because red inorganic color powder is composed of α-Fe_2_O_3_ (hematite) and yellow inorganic color powder mainly consists of α-FeOOH (goethite). Both of these are ferric ions (Fe^3+^), or what is commonly known as “rust”, and Fe^3+^ is considered a corrosion product (CP) in reinforced concrete (RC), which happens to give the colored mortar samples a natural look of a rust layer [33] while also enhancing resistance to ion migration on the sample surface and reducing the intrusion of noxious ions. Therefore, both red and yellow colored cement mortar samples have higher surface electrical resistivity than all of the other samples.

Surface electrical resistivity higher than 20 kΩ-cm means the sample has good compactness [34,35]. In this study, all of the colored mortar samples have surface electrical resistivities higher than 20 kΩ-cm at day 7, representing good surface compactness.

**Figure 13:**
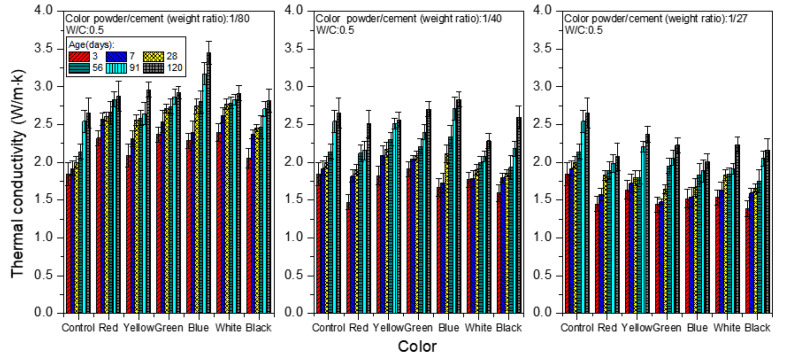
Thermal conductivity of colored mortar at W/C = 0.5 when weight ratios of color powder/cement at 1:80, 1:40, and 1:27 are added.

Figure 13 shows the thermal conductivity analysis of colored mortar at the W/C ratio of 0.5 when weight ratios of color powder/cement at 1:80, 1:40, and 1:27 were added. The thermal conductivity coefficients of the control group at different ages (day 3, day 28 and day 120) were 1.843, 2.001 and 2.650 W/m·K, respectively. For example, at day 28 with 1:80 inorganic color powder, the sample’s thermal conductivity coefficient was 2.453–2.771 W/m·K, 1.854–2.170 W/m·K with 1:40 inorganic color powder, and 1.647–1.835 W/m·K with 1:27 inorganic color powder. It was found that the thermal conductivity increases when 1:80 inorganic color powder is added, and increases with age, probably because inorganic nanoparticles themselves, in a moderate amount, have a higher thermal conductivity coefficient than cement; hence, they have the effect of boosting thermal conductivity. However, when inorganic color powder is increased to 1:40 and 1:27, the thermal conductivity trends downward, probably because inorganic color powder particles are porous, such that adding excessive color powder creates more air voids inside the colored cement mortar samples and results in a lower thermal conductivity.

### 3.4. Microstructure Analysis Results

**Figure 14:**
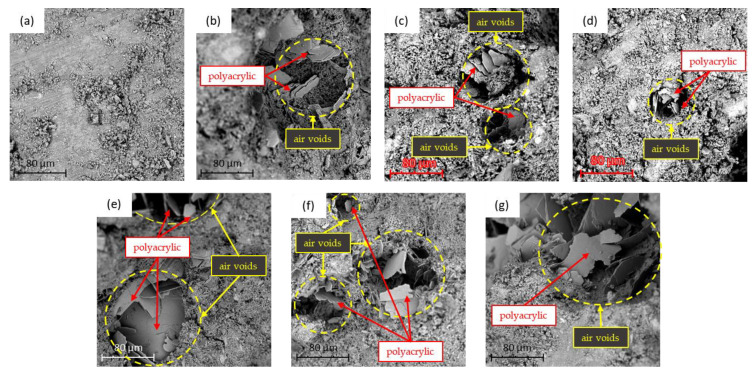
SEM images of the control group and colored cement mortar samples at day 28 with W/C = 0.5: (**a**) control, (**b**) red, (**c**) yellow, (**d**) green, (**e**) blue, (**f**) white, and (**g**) black.

Figure 14 shows the SEM image analysis of the control group and the colored cement mortar samples when the W/C ratio equals 0.5 at day 28. From the SEM analysis, it can be seen that the control group sample, with no color powder or polyacrylic emulsion added, shows a compact and condensed structure (as shown in Figure 14a). In contrast, the addition of color powder/cement at a weight ratio of 1:80 and polyacrylic emulsion/cement at a weight ratio of 1:5 for the sample is shown in the SEM images for Figure 14b–g; with 1:80 inorganic color powder and 1:5 polyacrylic emulsion added, many plate-shaped air voids can be seen. These are plate-shaped covers around the air voids after the polyacrylic emulsion solidifies, as air cannot be effectively expelled from the samples when the cement mortar is mixed with polyacrylic emulsion. Because of this, the colored cement mortar sample contains a large number of pores, and the mechanical strength of the sample is inferior to that of the control group. This is consistent with the aforementioned compressive and flexural strength analysis results. The addition of a polymer creates many air voids in the cement mortar, confirming the findings of Knapen et al. and Wang et al. [36,37]. In this regard, the use of a diluted solution [36], prewetting [38], or the addition of defoamer [39] may help to mitigate the formation of air voids. The aim of this study was to explore the influence of the addition of pigments to colored cement mortar; as such, defoaming was not performed.

## 4. Conclusions

In this study, colored cement mortar was made by adding inorganic color powder and polyacrylic emulsion to the mortar. The results show that the strength is reduced by the addition of inorganic color powder and acrylic emulsion to the cement mortar, while the control group (without adding any color) still had better mechanical strength and durability. However, with the addition of the weight ratio of color powder/cement at 1:80 inorganic color powder, polyacrylic emulsion/cement at 1:5, and W/C at 0.5 of acrylic emulsion, the color cement mortar has better performance. In the study of the fresh properties, colored cement mortar with added yellow inorganic color powder reduced the slump, and the slump flow was 7.6 and 23.83 cm, respectively, due to the greater internal friction of the particles, because the color powder has a relatively large surface area; this can be improved by adjusting the W/C ratio. In the study on the hardened properties, the results show that an appropriate W/C ratio and the amount of color powder added are critical factors determining the strength. An appropriate water/cement ratio (W/C = 0.5) ensures the adequate hydration reaction of the colored cement mortar, which boosts the sample’s structural strength. Adding excessive mixing water (W/C = 0.6) or porous inorganic color powder results in too many air voids in the samples that cannot easily be expelled, leading to the negative effect of lowered strength. Moreover, the sample prepared from the weight ratio of polyacrylic emulsion/cement at 1:5 makes the colored cement mortar weaker than the control group in terms of compressive strength, but outstanding results are seen in terms of flexural strength. When the weight ratio of color powder/cement is 1:80 at 91 days, the flexural strength of 19.9 MPa or more surpasses the control group when the polymer solidifies to become a film. Because adding excessive inorganic color powder creates more air voids, adding inorganic color powder (with a weight ratio of color/cement at 1:80) gives a better durability performance in terms of surface electrical resistivity and thermal conductivity. In the surface electrical resistivity analysis, the red and yellow colored cement mortar samples show outstanding performance; when aged for 120 days, the surface resistance reaches 62 and 52 kΩ-cm. An explanation is that both red and yellow inorganic color powders are ferric ions (Fe^3+^), which give the samples a natural rust layer that effectively reduces the intrusion of noxious ions, and a better result is seen in the red colored cement mortar sample at day 120. In terms of thermal conductivity analysis, because the added inorganic color powder itself has a higher thermal conductivity than cement, when added to cement mortar, the overall thermal conductivity of the colored cement mortar is improved.

## Figures and Tables

**Table 1:** Physical and chemical properties of the polyacrylic emulsion.

Physical Properties		Chemical Properties (wt.%)	
Solid content	50 ± 1%	Polyacrylic emulsion	50 ± 1
Viscosity	Under 500 cP	Water	50 ± 1
pH	9.0–10.0	-	-

**Table 2:** The physical properties of inorganic color powder.

Color Powder	Composition	Average Diameter (nm)	Surface Area (m^2^/g)	Pore Size (nm)	Thermal Conductivity (W/m·K)	Particle Shape
Red	α-Fe_2_O_3_ > 99%	120	6.78	43.8	16.0	Spherical
Yellow	α-FeOOH ≥ 73.6% Al(OH)_3_ ≤ 23.8%	100	63.4	8.19	-	Acicular
Green	Cr_2_O_2_ > 99%	300	2.12	49.2	5.17	Spherical
Blue	Na_7_Al_6_Si_6_O_24_S_3_ > 99%	500	5.26	30.5	-	Block
White	TiO_2_ > 96%	150	5.49	44.1	4.80	Spherical
Black	Fe_4_O_4_ > 99.3%	200	11.3	19.9	5.90	Spherical

**Table 3:** Mix ratios of colored mortar at W/C = 0.4, 0.5 and 0.6.

Samples	W/C	Cement (kg/m^3^)	Polyacrylic Emulsion (kg/m^3^)	Polyacrylic Emulsion/Cement (Weight Ratio)	Color Powder (kg/m^3^)	Color Powder/Cement (Weight Ratio)	Sand (kg/m^3^)	Water (kg/m^3^)
Control groups	0.4	624.26	-	-	-	-	1534.88	187.28
All colors of mortar		624.26	124.85	1:5	7.80	1:80		
					15.61	1:40		
					23.41	1:27		
Control groups	0.5	624.26	-	-	-		1534.88	249.71
All colors of mortar		624.26	124.85	1:5	7.80	1:80		
					15.61	1:40		
					23.41	1:27		
Control groups	0.6	624.26	-		-		1534.88	312.13
All colors of mortar		624.26	124.85	1:5	7.80	1:80		
					15.61	1:40		
					23.41	1:27		

Water/cement ratios: 0.4, 0.5, 0.6; Polyacrylic emulsion/cement: 1:5; Color powder/cement: 1:80, 1:40 and 1:27; Age: days 3, 7, 28, 56, 91 and 120; Color variables: control group, red, yellow, green, blue, white and black.

**Table 4:** UPV of colored mortar at W/C = 0.5 and under limewater curing when weight ratios of color powder/cement at 1:80, 1:40, and 1:27 are added.

Color	Polyacrylic Emulsion/Cement (Weight Ratio)	Color Powder/Cement (Weight Ratio)		Age (Day)			Unit (m/s)	
			3	7	28	56	91	120
Control	0	0	4348	4487	4545	4587	4623	4657
Red	1:5	1:80	3906	4007	4032	4049	4116	4269
		1:40	3704	3976	3985	4032	4049	4116
		1:27	3623	3789	3968	4016	4032	4049
Yellow	1:5	1:80	3954	4117	4167	4202	4238	4258
		1:40	3846	4000	4032	4117	4135	4220
		1:27	3806	3846	3881	3907	3969	3985
Green	1:5	1:80	3734	3923	4000	4016	4032	4039
		1:40	3690	3775	3954	3954	3969	4016
		1:27	3597	3637	3807	3922	3954	3986
Blue	1:5	1:80	3731	3923	4117	4117	4150	4202
		1:40	3704	3892	4004	4032	4098	4117
		1:27	3624	3804	3846	3922	4016	4032
White	1:5	1:80	3846	4065	4117	4132	4202	4238
		1:40	3664	3775	4032	4049	4065	4167
		1:27	3597	3760	3906	4032	4049	4116
Black	1:5	1:80	3789	3931	4117	4117	4132	4202
		1:40	3597	3775	4032	4048	4066	4082
		1:27	3571	3717	3954	3969	3985	4000

**Table 5:** Surface electrical resistivity of colored mortar at W/C = 0.5 when weight ratios of color powder/cement at 1:80, 1:40, and 1:27 are added.

Color	Polyacrylic Emulsion/Cement (Weight Ratio)	Color Powder/Cement (Weight Ratio)		Age (Day)			Unit (kΩ-cm)	
			3	7	28	56	91	120
Control	0	0	14	19	22	29	30	47
Red	1:5	1:80	17	27	35	36	43	62
		1:40	16	24	30	34	35	41
		1:27	14	22	29	32	35	56
Yellow	1:5	1:80	27	28	42	44	46	52
		1:40	19	23	36	39	40	41
		1:27	17	21	33	36	38	41
Green	1:5	1:80	24	25	31	37	38	43
		1:40	21	24	30	33	34	40
		1:27	19	23	25	29	30	36
Blue	1:5	1:80	17	24	41	44	48	51
		1:40	16	23	29	35	37	39
		1:27	15	23	28	30	35	38
White	1:5	1:80	21	23	31	35	39	40
		1:40	20	22	29	31	33	40
		1:27	19	21	24	29	31	39
Black	1:5	1:80	22	24	32	36	40	43
		1:40	20	21	28	35	39	40
		1:27	18	21	28	22	35	36

## Data Availability

Data sharing is not applicable for this article.
